# Body mass index and survival after breast cancer diagnosis in Japanese women

**DOI:** 10.1186/1471-2407-12-149

**Published:** 2012-04-17

**Authors:** Masaaki Kawai, Yuko Minami, Yoshikazu Nishino, Kayoko Fukamachi, Noriaki Ohuchi, Yoichiro Kakugawa

**Affiliations:** 1 Division of Community Health, Tohoku University Graduate School of Medicine, 2-1 Seiryo-machi, Aoba-ku, Sendai, Miyagi, 980-8575, Japan; 2 Division of Cancer Epidemiology and Prevention, Miyagi Cancer Center Research Institute, 47-1 Nodayama, Medeshima-Shiode, Natori, Miyagi, 981-1293, Japan; 3 Department of Breast Oncology, Miyagi Cancer Center Hospital, 47-1 Nodayama, Medeshima-Shiode, Natori, Miyagi, 981-1293, Japan; 4 Department of Surgical Oncology, Tohoku University Graduate School of Medicine, 1-1 Seiryo-machi, Aoba-ku, Sendai, Miyagi, 980-8574, Japan

**Keywords:** Breast cancer, Survival, Body mass index, Hormone receptor, Menopausal status

## Abstract

**Background:**

Body mass index (BMI) may be an important factor affecting breast cancer outcome. Studies conducted mainly in Western countries have reported a relationship between higher BMI and a higher risk of all-cause death or breast cancer-specific death among women with breast cancer, but only a few studies have been reported in Japan so far. In the present prospective study, we investigated the associations between BMI and the risk of all-cause and breast cancer-specific death among breast cancer patients overall and by menopausal status and hormone receptor status.

**Methods:**

The study included 653 breast cancer patients admitted to a single hospital in Japan, between 1997 and 2005. BMI was assessed using a self-administered questionnaire. The patients were completely followed up until December, 2008. Hazard ratios (HRs) and 95% confidence intervals (CIs) were estimated according to quartile points of BMI categories, respectively: <21.2, ≥21.2 to <23.3 (reference), ≥23.3 to <25.8 and ≥25.8 kg/m^2^.

**Results:**

During the follow-up period, 136 all-cause and 108 breast cancer-specific deaths were observed. After adjustment for clinical and confounding factors, higher BMI was associated with an increased risk of all-cause death (HR = 2.61; 95% CI: 1.01–6.78 for BMI ≥25.8 vs. ≥21.2 to <23.3 kg/m^2^) among premenopausal patients. According to hormonal receptor status, BMI ≥25.8 kg/m^2^ was associated with breast cancer-specific death (HR = 4.95; 95% CI: 1.05–23.35) and BMI <21.2 kg/m^2^ was associated with all-cause (HR = 2.91; 95% CI: 1.09–7.77) and breast cancer-specific death (HR = 7.23; 95% CI: 1.57–33.34) among patients with ER + or PgR + tumors. Analysis by hormonal receptor status also showed a positive association between BMI and mortality risk among patients with ER + or PgR + tumors and with BMI ≥21.2 kg/m^2^ (p for trend: 0.020 and 0.031 for all-cause and breast cancer-specific death, respectively).

**Conclusions:**

Our results suggest that both higher BMI and lower BMI are associated with an increased risk of mortality, especially among premenopausal patients or among patients with hormonal receptor positive tumors. Breast cancer patients should be informed of the potential importance of maintaining an appropriate body weight after they have been diagnosed.

## Background

Many previous epidemiologic studies have demonstrated that higher body mass index (BMI) is associated with an increased risk of postmenopausal breast cancer, whereas it is associated with a reduced risk of premenopausal breast cancer [1]. Furthermore, some studies conducted mainly in Western countries have found associations between higher BMI and a higher risk of all-cause death [2-10] or breast cancer-specific death [6,11,12] among women with breast cancer, although other studies have found no such association [13-16]. As various inconsistencies have been reported across menopausal status between BMI and survival among premenopausal [2,4,8,12,17-21] and postmenopausal women [5,8,11,12,21], it is important to stratify menopausal status in order to adequately assess the relationship between BMI and mortality of breast cancer patients.

In adipose tissue, conversion of androgens to estrogens by aromatase occurs [22]. Estrogen accelerates breast tumor growth via the estrogen receptor. Breast tumors have estrogen or progesterone receptors, and tumor subtypes defined by these receptors may represent biologically different entities [23,24] and influence the survival of patients. Therefore it seems important to consider tumor subtypes when evaluating the relationship between BMI and mortality due to breast cancer, and in fact several studies have already investigated the effects of tumor subtype in terms of hormone receptor status [2,4,9,10,13,14,20].

In Japan, two previous studies have assessed the relationship between BMI and survival in breast cancer patients [25,26]. However, those studies were small in scale and controlled for only a few known risk factors. Only one previous study has addressed this issue in terms of menopausal status [26], but no attempt has yet been made to do so in terms of hormone receptor status.

In the present study, therefore, we investigated the relationship between BMI and the risk of all-cause death and breast cancer-specific death among breast cancer patients in terms of menopausal status and also hormone receptor status using a hospital-based prospective cohort study. Some known risk factors, tumor stage, and data on the therapy used for breast cancer were taken into account as covariates. Analyses stratified according to menopausal and hormone receptor status were performed, along with analysis of the patients overall.

## Methods

### Study subjects

Between January 1997 and December 2005, 718 female patients aged 29 years or over were newly diagnosed as having breast cancer at the Miyagi Cancer Center Hospital (MCCH). All of these patients were requested to complete a questionnaire upon initial admission. After diagnosis, their details were entered into the hospital-based cancer registry and the patients were followed up. This cancer registry recorded clinical and pathological findings and information on antineoplastic treatments for all patients with cancer admitted to the MCCH. The MCCH is located in Natori City, situated in the southern part of Miyagi Prefecture. It has 383 administrative beds, and functions as both a general hospital and a comprehensive research institute for both all types of cancer and benign diseases.

Among the 718 newly diagnosed breast cancer patients, 664 (92.5%) completed the questionnaire. After excluding 7 patients with a history of cancers other than breast cancer, the 657 remaining patients were included in the present study, which was approved by the ethical review board of Miyagi Cancer Center.

### Questionnaire and clinical information

In January 1997, we began a survey in connection with the present study. Information on lifestyle and personal history was collected from all patients using a self-administered questionnaire, which was distributed to patients on the day of their reservation for initial admission to the MCCH, i.e., 10–15 days before admission, and collected by nurses on the actual admission day. Details of the questionnaire survey have already been described elsewhere [27,28].

The questionnaire covers items on demographic characteristics, current height and weight, family histories of cancer and other diseases, general lifestyle factors before the development of current symptoms including history of smoking, menopausal status, and comorbidity of other diseases.

Clinical information including tumor stage and treatment, such as chemotherapy, radiation therapy and endocrine therapy, was obtained from the MCCH hospital-based cancer registry. Information on hormone receptor status, i.e. expression of the estrogen receptor (ER) and progesterone receptor (PgR), was extracted from medical records. To measure ER and PgR status, enzyme immunoassay (EIA) was used in the early period of the study. After mid-2003, immunohistochemistry (IHC) was conducted. The cut-off point for receptor positivity in the EIA was 14 fmol/mg for ER and 13 fmol/mg for PgR. In the IHC assay, a histology score (HSCORE) of ≥20 for ER and one of ≥6 for PgR were evaluated as positive [29]. The concordance between the two assays was 94.3% for ER and 100% for PgR in the laboratory of the MCCH [29]. Receptor status was unknown for ER in 69 cases (10.5%), PgR in 80 (12.2%) cases, and both in 69 (10.5%) cases. 392 (59.7%) cases were ER + and 318 (48.4%) were PgR + .

### Ascertainment of exposures and follow-up

At the MCCH, initial therapy is administered after admission in principal. Therefore, data on weight and height collected using the questionnaire was considered to be pretreatment data. BMI was calculated as weight divided by the square of current height (kg/m^2^). Height and weight were measured by medical staff in a subsample (n = 315) of our study at the time of initial hospital admission. The self-reported height and weight data were highly correlated with the measured data (correlation coefficient: 0.94 for height and 0.96 for weight). Four patients for whom BMI values were missing were excluded, leaving a final total of 653 patients for analysis. We stratified the patients according to BMI quartile points: <21.2 kg/m^2^, ≥21.2 kg/m^2^ to <23.3 kg/m^2^, ≥23.3 kg/m^2^ to <25.8 kg/m^2^ and ≥25.8 kg/m^2^. The BMI category ≥21.2 kg/m^2^ to <23.3 kg/m^2^ was selected as the reference.

Follow-up was performed by reference to the MCCH Cancer Registry up to December 31, 2008. Active follow-up was conducted by accessing hospital visit records, resident registration cards and permanent domicile data. Information on the dates and causes of death was obtained with permission from the Ministry of Justice. During the study period, no subject was lost to follow-up.

### Statistical analysis

The end point of our analysis was all-cause death and breast cancer-specific death according to the International Classification of Disease for Oncology, Tenth Edition (ICD-10). Survival time was calculated for each patient from the date of diagnosis to the date of death or the end of follow-up (December 31, 2008).

The Cox proportional hazards model was used to estimate hazard ratios (HRs) and 95% confidence intervals (CIs) for all-cause death and breast cancer-specific death in relation to BMI [30]. Tests for trend were employed in the Cox model for all BMI categories and for ≥21.2 kg/m^2^ respectively, because we expected the overall relationship of BMI to mortality to be U-shaped rather than linear (i.e., we expected women with BMI <21.2 kg/m^2^ have higher mortality than the reference category). We considered the following variables to be potential confounders: age, tumor stage (in situ or localized, local invasion, lymph node metastasis, distant metastasis), hormone receptor status (ER + or PgR+, ER-/PgR-), radiation therapy (no, yes), chemotherapy (no, yes), endocrine therapy (no, yes) and comorbidities (no, yes). Comorbidities included hypertension, ischemic heart disease, stroke and diabetes mellitus. Smoking (current, past, never), family history of breast cancer in mother or sister (no, yes), and physical activity (almost no, more than one hour per week, missing), some of which have already been established as risk factors for breast cancer, were also considered to be adjusted for [31-33]. Missing values for confounders were treated as an additional variable category, and included in the model.

Separate analyses were conducted after dividing the patients according to premenopausal or postmenopausal status, along with analysis of the patients overall. Stratification according to hormonal receptor status was also performed. To evaluate heterogeneity of the associations between BMI and all-cause death and breast cancer-specific death across menopausal status (premenopausal vs. postmenopausal) and hormone receptor status (ER + or PgR + vs. ER-/PgR-), interaction terms (BMI * menopausal status, BMI * hormone receptor status) were tested. Likelihood ratio tests were used to assess the significance of heterogeneity by comparing the model including the interaction term to the main-effects model. Menopause was defined as the cessation of menstrual periods due to natural or other reasons, including surgery. With regard to menopause due to other reasons, we were unable to obtain any information about history of oophorectomy; therefore, patients 44–57 years of age (defined as the mean age at natural menopause ±2 SD) were regarded as having unknown menopausal status.

Results were regarded as significant if the two-sided P values were <0.05. All statistical analyses were performed using the SAS software package (version 9.2; SAS Institute, Cary, NC).

## Results

During a median follow-up period of 5.85 years, 136 all-cause and 108 breast cancer-specific deaths were observed. The characteristics of the patients at the time of breast cancer diagnosis are shown in Table 1. Heavier patients tended to have hormonal receptor-positive tumors. With regard to hormone receptor status, 410 (62.8%) cases were ER + or PgR+, and 174 (26.6%) were ER-/PgR-. Women with higher BMI were more likely to be older, to be postmenopausal, to exercise more, to have more comorbidities, and to have hormone receptor-positive tumors.

**Table 1:** Characteristics of the study cohort

		**BMI**				**Total**
		**< 21.2**	≥**21.2 to< 23.3**	≥**23.3 to< 25.8**	≥**25.8**	
Age (years) mean ± S.D.		53.7 ± 13.4	55.2 ± 11.2	58.9 ± 12.0	60.0 ± 11.9	57.0 ± 12.4
Person-years		963.0	1052.4	1020.1	997.3	4032.8
Patients (n)		163	166	161	163	653
All-cause death (n)		42	27	29	38	136
Breast cancer-specific death (n)		34	21	26	27	108
Smoking (%)						
	Never	72.4	78.9	85.7	82.2	79.8
	Current or Past	25.2	17.5	12.4	14.1	17.3
	Missing	2.5	3.6	1.9	3.7	2.9
Stage (%)						
	In situ or Locarized	39.9	43.4	34.2	39.3	39.2
	Lymph node Metastasis	30.7	34.9	41.6	35.6	35.7
	Local Invasion	10.4	6.6	9.9	8.0	8.7
	Distant Metastasis	1.8	2.4	5.0	3.1	3.1
	Missing	17.2	12.7	9.3	14.1	13.3
Hormone receptor (%)						
	ER + or PgR+	58.9	57.8	65.2	69.3	62.8
	ER-/PgR-	28.2	30.1	25.5	22.7	26.6
	Missing	12.9	12.0	9.3	8.0	10.6
Radiation therapy (%)						
	No	77.3	78.3	86.3	83.4	81.3
	Yes	22.7	21.7	13.7	16.6	18.7
Chemotherapy (%)						
	No	74.8	76.5	75.2	78.5	76.3
	Yes	25.2	23.5	24.8	21.5	23.7
Endocrine therapy (%)						
	No	75.5	75.9	79.5	71.2	75.5
	Yes	24.5	24.1	20.5	28.8	24.5
	No	95.1	92.8	90.7	86.5	91.3
	Yes	4.9	7.2	9.3	13.5	8.7
Menopausal status (%) ^a^						
	Premenopausal	54.6	44.0	39.1	31.9	42.4
	Postmenopausal	41.1	51.8	57.8	66.3	54.2
	Missing		4.2	3.1	1.8	3.4
Physical activity (%)						
	Almost no	51.5	53.0	45.3	46.6	49.2
	More than one hour per week	43.6	39.8	46.0	50.3	44.9
	Missing	4.9	7.2	8.7	3.1	6.0
Comorbidities (%) ^b^						
	No	86.5	80.1	71.4	67.5	76.4
	Yes	13.5	19.9	28.6	32.5	23.6

^a^ Menopause was defined as the cessation of menstrual periods due to natural or other reasons including surgery.

^b^ Includes hypertension/ischemic heart disease/stroke/diabetes mellitus.

During a median follow-up period of 5.85 years, 136 all-cause and 108 breast cancer-specific deaths were observed.

Table 2 shows the association of BMI with all-cause death. Compared to women with BMI ≥21.2 to <23.3 kg/m^2^, those with BMI <21.2 kg/m^2^ were shown to have a higher risk of death by age-adjusted analysis (HR = 1.73, 95% CI: 1.07–2.80), but not by multivariate-adjusted analyses (1.60, 0.97–2.63). No dose–response relationship was observed between BMI and all-cause death (multivariate-adjusted p for trend = 0.59). Analysis limited to women with BMI ≥21.2 kg/m^2^ also demonstrated no dose–response relationship (multivariate-adjusted p for trend = 0.11). Stratification by menopausal status yielded inconsistent results. BMI had no significant association with all-cause death among postmenopausal women, whereas a significantly increased risk of all-cause death was found among premenopausal obese women (BMI ≥25.8 kg/m^2^) in both age-adjusted (2.49, 1.03–6.03) and multivariate-adjusted analyses (2.61, 1.01–6.78). For premenopausal women with BMI ≥21.2 kg/m^2^, trend test demonstrated a marginal dose–response relationship between BMI and all-cause death (multivariate-adjusted p for trend = 0.059). The trends were not significantly different between premenopausal and postmenopausal women with BMI ≥21.2 kg/m^2^ (P for heterogeneity of trends = 0.11).

**Table 2:** HR (95%CI) of all-cause death associated with BMI overall and by menopausal status

		**BMI**	**Patients**	**Person-years**	**All-cause death**	**Age-adjusted**			**Multivariate-adjusted**		
						**HR**	**95% CI**		**HR**	**95% CI**	
All											
		<21.2	163	963.0	42	1.73	1.07 - 2.80		1.60	0.97 - 2.63	
		≥21.2 to <23.3	166	1052.4	27	1.00 (reference)			1.00 (reference) ^a^		
		≥23.3 to <25.8	161	1020.1	29	1.03	0.61 - 1.75		0.88	0.51 - 1.51	
		≥25.8	163	997.3	38	1.37	0.83 - 2.25		1.46	0.87 - 2.44	
	p for trend							0.35			0.59
	p for trend in women with BMI ≥21.2							0.18			0.11
Premenopausal											
		<21.2	89	556.1	18	2.04	0.88 - 4.69		1.75	0.71 - 4.29	
		≥21.2 to <23.3	73	510.2	8	1.00 (reference)			1.00 (reference) ^b^		
		≥23.3 to <25.8	63	391.6	11	1.74	0.70 - 4.35		1.61	0.63 - 4.11	
		≥25.8	52	319.8	13	2.49	1.03 - 6.03		2.61	1.01 - 6.78	
	p for trend							0.52			0.29
	p for trend in women with BMI ≥21.2							0.05			0.059
Postmenopausal											
		<21.2	67	371.6	20	1.56	0.82 - 2.98		0.93	0.47 - 1.86	
		≥21.2 to <23.3	86	500.7	17	1.00 (reference)			1.00 (reference) ^b^		
		≥23.3 to <25.8	93	589.2	16	0.74	0.37 - 1.47		0.45	0.21 - 0.94	
		≥25.8	108	670.7	22	0.93	0.49 - 1.75		0.72	0.36 - 1.45	
	p for trend							0.086			0.2
	p for trend in women with BMI ≥21.2							0.91			0.71
Pre v Post p for heterogeneity of trends								0.13			0.24
Pre v Post p for heterogeneity of trends in women with BMI ≥21.2								0.09			0.11

^a^ Adjusted by age, stage (in situ or localized, lymph node metastasis, local invasion, distant metastasis, missing), hormone receptor (ER + or PgR+, ER-/PgR-, missing), radiation therapy (no, yes), chemotherapy (no, yes), endocrine therapy (no, yes), smoking (current, past, never, missing), family history of breast cancer in father, mother, brother or sister (no, yes), menopausal status (premenopausal, postmenopausal, missing), physical activity (almost no, more than one hour per week, missing) and comorbidities (no, yes).

^b^ Adjusted by age, stage, hormone receptor, radiation therapy, chemotherapy, endocrine therapy, smoking, family history of breast cancer in father, mother, brother or sister, physical activity and comorbidities.

An increased risk of all-cause death was found among premenopausal women with BMI ≥25.8 kg/m^2^. Trend test for premenopausal women with BMI ≥21.2 kg/m^2^ also showed a marginal dose–response relationship.

With regard to breast cancer-specific death, age-adjusted analysis and multivariate-adjusted analysis showed that women with BMI <21.2 kg/m^2^ were not at higher risk (Table 3). No dose-response relationship between BMI and breast cancer-specific death was found.

**Table 3:** HR (95%CI) of breast cancer-specific death associated with BMI overall and by menopausal status

		**BMI**	**Patients**	**Person-years**	**Breast cancer- specific death**	**Age-adjusted**			**Multivariate-adjusted**		
						**HR**	**95% CI**		**HR**	**95% CI**	
All											
		<21.2	163	963.0	34	1.69	0.98 - 2.92		1.59	0.90 - 2.81	
		≥21.2 to <23.3	166	1052.4	21	1.00 (reference)			1.00 (reference) ^a^		
		≥23.3 to <25.8	161	1020.1	26	1.32	0.74 - 2.35		1.20	0.66 - 2.17	
		≥25.8	163	997.3	27	1.40	0.79 - 2.49		1.46	0.81 - 2.64	
	p for trend							0.64			0.87
	p for trend in women with BMI ≥21.2							0.20			0.18
Premenopausal											
		<21.2	89	556.1	15	1.60	0.68 - 3.80		1.22	0.47 - 3.14	
		≥21.2 to <23.3	73	510.2	8	1.00 (reference)			1.00 (reference) ^b^		
		≥23.3 to <25.8	63	391.6	11	1.77	0.71 - 4.41		1.62	0.63 - 4.20	
		≥25.8	52	319.8	10	1.95	0.77 - 4.96		1.68	0.61 - 4.65	
	p for trend							0.48			0.34
	p for trend in women with BMI ≥21.2							0.18			0.51
Postmenopausal											
		<21.2	67	371.6	15	1.89	0.87 - 4.11		1.22	0.52 - 2.86	
		≥21.2 to <23.3	86	500.7	11	1.00 (reference)			1.00 (reference) ^b^		
		≥23.3 to <25.8	93	589.2	13	1.09	0.49 - 2.45		0.79	0.32 - 1.93	
		≥25.8	108	670.7	14	1.02	0.46 - 2.26		1.03	0.43 - 2.50	
	p for trend							0.15			0.56
	p for trend in women with BMI ≥21.2							0.86			0.45
Pre v Post p for heterogeneity of trends								0.13			0.27
Pre v Post p for heterogeneity of trends in women with BMI ≥21.2								0.29			0.80

^a^ Adjusted by age, stage (in situ or localized, lymph node metastasis, local invasion, distant metastasis, missing), hormone receptor (ER + or PgR+, ER-/PgR-, Missing), radiation therapy (no, yes), chemotherapy (no, yes), endocrine therapy (no, yes), smoking (current, past, never, missing), family history of breast cancer in father, mother, brother or sister (no, yes), menopausal status (premenopausal, postmenopausal, missing), physical activity (almost no, more than one hour per week, missing) and comorbidities (no, yes).

^b^ Adjusted by age, stage, hormone receptor, radiation therapy, chemotherapy, endocrine therapy, smoking, family history of breast cancer in father, mother, brother or sister, physical activity and comorbidities.

No dose–response relationship between BMI and breast cancer-specific death was found.

Analysis stratified by hormonal receptor status demonstrated differences in the risk of death across strata for ER/PR status (Table 4). Among women with ER + or PgR + tumors, BMI was significantly associated with both all-cause (multivariate-adjusted p for trend = 0.02) and breast cancer-specific death (multivariate-adjusted p for trend = 0.031) if the women had a BMI of ≥21.2 kg/m^2^. Heavier women (≥25.8 kg/m^2^) with ER + or PgR + tumors showed a higher risk of breast cancer-specific death (4.95, 1.05–23.35). BMI <21.2 kg/m^2^ carried a higher risk of all-cause (2.91, 1.09–7.77) and breast cancer-specific death (7.23, 1.57–33.34) compared to women with BMI ≥21.2 to <23.3 kg/m^2^. No significant association between BMI and all-cause and breast cancer-specific death was found for ER-/PgR- tumors. For all-cause and breast cancer-specific death, the trends were not significantly different between ER + or PgR + and ER-/PgR- women with BMI ≥21.2 kg/m^2^ (P for heterogeneity of trends = 0.10 and 0.13, respectively).

**Table 4:** HR (95%CI) of all-cause and breast cancer-specific death associated with BMI by hormone receptor status

		**BMI**	**Patients**	**Person-years**	**All-cause death**				**Breast cancer-specific death**			
					**Number of death**	**HR ^a^**	**95% CI**		**Number of death**	**HR ^a^**	**95% CI**	
ER + or PgR+												
		<21.2	96	581.5	18	2.91	1.09 - 7.77		16	7.23	1.57 - 33.34	
		≥21.2 to <23.3	96	612.5	6	1.00 (reference)			2	1.00 (reference)		
		≥23.3 to <25.8	105	670.5	12	1.16	0.41 - 3.24		9	3.31	0.67 - 16.41	
		≥25.8	113	708.9	21	2.49	0.96 - 6.47		12	4.95	1.05 - 23.35	
	p for trend							0.85				0.57
	p for trend in women with BMI ≥21.2							0.02				0.031
ER-/PgR-												
		<21.2	46	295.2	14	0.95	0.41 - 2.20		10	0.90	0.35 - 2.29	
		≥21.2 to <23.3	50	350.7	12	1.00 (reference)			11	1.00 (reference)		
		≥23.3 to <25.8	41	265.0	13	0.72	0.30 - 1.75		13	0.88	0.35 - 2.21	
		≥25.8	37	241.9	11	0.94	0.38 - 2.33		9	1.00	0.38 - 2.64	
	p for trend							0.77				0.91
	p for trend in women with BMI ≥21.2							0.96				0.98
ER + or PgR + v ER-/PgR- p for heterogeneity of trends								0.80				0.61
ER + or PgR + v ER-/PgR- p for heterogeneity of trends in women with BMI ≥21.2								0.10				0.13

^a^ Adjusted by age, stage (in situ or localized, lymph node metastasis, local invasion, distant metastasis, missing), radiation therapy (no, yes), chemotherapy (no, yes), endocrine therapy (no, yes), smoking (current, past, never, missing), family history of breast cancer in father, mother, brother or sister (no, yes), menopausal status (premenopausal, postmenopausal, missing), physical activity (almost no, more than one hour per week, missing) and comorbidities (no, yes).

Among women with ER + or PgR + tumors, BMI was significantly associated with both all-cause and breast cancer-specific death in those with BMI ≥21.2 kg/m^2^, and lighter (BMI <21.2 kg/m^2^) women also had a higher risk of all-cause and breast cancer-specific death.

## Discussion

This study demonstrated that higher BMI was significantly associated with all-cause death among premenopausal patients after adjustment for clinical and known factors that are associated with the mortality risk of breast cancer patients. Analysis stratified according to hormonal receptor status showed that higher and lower BMI were associated with increased risks of all-cause and breast cancer-specific death only for patients with ER + or PgR + tumors. Previous studies that investigated the relationship between BMI and outcome in Japanese breast cancer patients considered only a few known risk factors as covariates, included only a small number of cases, and did not assess hormone receptor status [25,26]. Our study is of importance in having assessed the relationship between BMI and all-cause or breast cancer-specific death by taking into account multiple risk factors for breast cancer, in addition to menopausal status and hormone receptor status, in Japanese women.

Our results demonstrated that higher BMI was significantly associated with all-cause death among premenopausal patients, and were consistent with several previous observational studies of premenopausal or younger women that demonstrated poorer overall survival with increased BMI [2,4,17,18,20]. A meta-analysis including 43 studies showed that the effect of obesity on higher all-cause or breast cancer-specific death was larger among premenopausal than among postmenopausal women [21]. Our present results demonstrated that the effect of higher BMI was greater for all-cause death than for breast cancer-specific death (HR = 2.61; 95% CI: 1.01–6.78 for BMI ≥25.8 kg/m^2^ for all-cause death; HR = 1.68; 95% CI: 0.61–4.65 for BMI ≥25.8 kg/m^2^ for breast cancer-specific death). One possibility is that women with higher BMI have poorer overall survival because of a higher risk of comorbidities. Therefore, we reanalyzed the data after excluding patients who had comorbidities. Within the limited statistical power, the effect of higher BMI was significant for all-cause death among premenopausal patients (HR = 3.42; 95% CI: 1.23–9.47 for BMI ≥25.8 kg/m^2^ for all-cause death, p for trend for BMI ≥21.2 kg/m^2^ = 0.0068) and not significant for breast cancer-specific death. These were perhaps potential mediators of the adverse effect of higher BMI in premenopausal breast cancer patients, independent of comorbidities.

In the present study, an association of higher BMI with poorer outcome was seen in women with ER + or PgR + tumors. This result is consistent with previous studies that have indicated an association of higher BMI with poorer outcome, being especially pronounced among women with hormone receptor-positive tumors [9,10]. Several hypotheses to explain why obese breast cancer patients show poorer survival can be considered. Firstly, there may be differences in sensitivity to estrogen among tumors with different types of hormone receptors. A previous study found that hormone receptor-positive tumors showed a better response to endocrine therapy than ER-/PgR- tumors [34], indicating that ER + or PgR + tumors are the most sensitive to estrogen hormone. Secondly, it has been postulated that higher estrogen concentrations may confer increased biological aggressiveness on hormone receptor-positive tumors, as BMI is directly related to circulating estrogen levels [22,35,36]. Thirdly, higher BMI is associated with upregulation of a number of cellular proliferation pathways [37]. Consequently, obesity might lead to an increase of tumor cell proliferation and metastasis through undefined adipokine effects on tumor cells [17]. For example, leptin, an adipocytokine, is produced mainly by adipose tissue and is known to act as a cancer growth factor [38], as well as promoting angiogenesis and potentially stimulating the growth of breast cancer cells, thus possibly leading to reduced patient survival [39].

Our present multivariate-adjusted analysis showed that BMI <21.2 kg/m^2^, i.e. low BMI, was associated with elevated risks of both all-cause and breast cancer-specific death among women with ER + or PgR + tumors. The relationship between low BMI and higher cancer mortality risk might be at least partly explained by the presence of circulating tumor cells (CTCs) in the peripheral blood of patients [40]. CTCs are derived from clones in the primary tumor [41] and are thought to become scattered to various organs, leading to the development of distant metastasis [42]. In patients showing chronic undernutrition, cytokine reactions and subsequent activation of the immune system are compromised, which might affect the tumor-immune system interaction in distant organs [43]. In this study, the BMI <21.2 kg/m^2^ category might have included undernourished patients as well as properly nourished, naturally lean patients. This may have partly contributed to the increased risk of all-cause and breast cancer-specific death. Another reason for the relationship between the BMI <21.2 kg/m^2^ category and higher risk of cancer mortality might have been the slightly higher proportion of patients with advanced-stage breast cancer. Therefore, we attempted to analyze the data by omitting cases of advanced breast cancer. However, this analysis yielded almost the same results (data not shown).

The major strengths of the present study were that no subject was lost to follow-up during the study period. The MCCH Cancer Registry conducts active follow-up by accessing hospital visit records, resident registration cards and permanent domicile data. In cases of death occurring outside the hospital, information on the date and cause of death was obtained with permission from the Ministry of Justice. Another strength was the relatively low proportion of patients for whom data on hormone receptor status were missing (10.6%). In previous studies, the proportion of patients for whom data on ER and/or PgR status were missing ranged from 5.0% to 48.1% [2,4,9,10]. Distribution of receptor status for ER and PgR was roughly the same as those in previous studies which investigated 3,089 patients from ten hospitals in Japan [44]. A further strength was that it gave consideration not only to clinical stages but also to treatments such as chemotherapy, endocrine therapy and radiation therapy from an epidemiological viewpoint.

Several limitations of our study should also be considered. First, although BMI has been accepted as an index of obesity, it cannot be used to identify the distributions of fat and muscle tissue. Second, we used self-reported BMI at the baseline, and there may have been a misclassification of exposure due to self-reported weight and height. However, the self-reported current height and weight data were highly correlated with measured data, and therefore any possible bias was likely small. Third, stratification by hormone receptor status may have resulted in false positive or false negative results. The 95% CIs were wide for HRs by hormone receptor status, suggesting that statistical power might be limited because of relatively small number of patients and all-cause and breast cancer-specific deaths. To obtain reliable results with this stratification, subsequent recruitment of patients and follow-up will be required. Fourth, the generalizability of our results to the Japanese population as whole may be limited because our study was conducted among a population living in a rural area. More studies are needed to verify our results instead of to assess the generalizability.

## Conclusions

In conclusion, being obese is a risk factor for all-cause death in premenopausal women and a risk factor for all-cause and breast cancer-specific death in patients with ER + or PgR + tumors. Lower BMI is associated with higher all-cause and breast cancer-specific death in patients with ER + or PgR + tumors. As higher and lower BMI are directly related to mortality [45], it is important to maintain an appropriate body weight for height.

## Abbreviations

MCCH, Miyagi Cancer Center Hospital; ER, Estrogen receptor; PgR, Progesterone receptor; EIA, Enzyme immunoassay; IHC, Immunohistochemistry; HSCORE, Histology score; HR, Hazard ratio; CI, Confidence interval; CTC, Circulating tumor cell.

## Competing interests

The authors declare that they have no competing interests.

## Authors' contributions

MK, YM, YN, YK participated in the design of the study. MK, YM participated in the statistical analysis of the data. MK, YM, KF, YN, NO, YK drafted the manuscript. MK, YM, KF, YN, YK participated in the collection of the data. All authors read and approved the final manuscript.

## Pre-publication history

The pre-publication history for this paper can be accessed here:

http://www.biomedcentral.com/1471-2407/12/149/prepub
